# The II–I Phase Transition Behavior of Butene-1 Copolymers with Hydroxyl Groups

**DOI:** 10.3390/polym13081315

**Published:** 2021-04-16

**Authors:** Yuanyuan Li, Tao Li, Wei Li, Yahui Lou, Liyuan Liu, Zhe Ma

**Affiliations:** 1 Tianjin Key Laboratory of Composite and Functional Materials and School of Materials Science and Engineering, Tianjin University, Tianjin 300072, China; yyli@tju.edu.cn (Y.L.); litao0416@tju.edu.cn (T.L.); weili_wq@tju.edu.cn (W.L.); yhlou@tju.edu.cn (Y.L.); 2 Center for Terahertz Waves and College of Precision Instrument and Optoelectronics Engineering, Tianjin University, Tianjin 300072, China

**Keywords:** butene-1 copolymer, hydroxyl group, phase transition, crystallization

## Abstract

The crystallization and II–I phase transition of functionalized polybutene-1 with hydroxyl groups were investigated by differential scanning calorimetry. The results show that the incorporated hydroxyl groups increase the nucleation density but decrease the growth rate in melt crystallization. Interestingly, for the generated tetragonal form II, the presence of polar hydroxyl groups can effectively accelerate the phase transition into the thermodynamically stable modification of trigonal form I, especially with stepwise annealing and high incorporation. Using stepwise annealing, II–I phase transition was enhanced by an additional nucleation step performed at a relatively low temperature, and the optimal nucleation temperature to obtain the maximum transition degree was ‒10 °C, which is independent from the content of hydroxyl groups. Furthermore, the accelerating effect of hydroxyl groups on the II–I transition kinetics can be increased by reducing the crystallization temperature when preparing form II crystallites. These results provide a potential molecular design approach for developing polybutene-1 materials.

## 1. Introduction

Polyolefin has many outstanding properties such as good chemical resistance, light weight, easy processing ability, strong mechanical strength, and low cost. In recent years, increasing attention has been paid to the functionalization of polyolefin to obtain diverse molecular structures and to improve the performance of polyolefin materials.

For this purpose, incorporating additional polar functional groups into the original nonpolar polyolefin chains is a very effective strategy [1,2,3,4]. Yuan et al. synthesized functionalized polypropylenes containing hydroxyl groups and found that the dielectric constant was greatly improved owing to the alignment of polarizable pendant hydroxyl groups under an electric field [5,6,7,8]. Dai et al. prepared and studied a thermoplastic elastomer of polar functionalized polyethylene. It was found that the presence of polar monomers improved the surface performance, adhesion, and compatibility without significantly decreasing the elastic recovery [9]. Recently, Shang et al. successfully synthesized amino-containing polypropylene. Their results show that the presence of the amino groups improved the surface properties and thermal stability of the materials [10]. Although there have been efforts to modify the ultimate structure, the correlation between the polar functional groups and the crystallite structure is still far from conclusive. Clarity is urgently required for efficient design of the molecular structure and ultimate physical properties.

Polybutene-1 (PB-1) is an important type of polyolefin material that possesses some excellent mechanical properties such as high toughness and creep resistance [11]. As a polymorphic polymer, PB-1 chains can be packed into three types of crystalline modifications with different helix conformations, which determine the final mechanical performance [11,12,13,14,15,16]. When the polymer undergoes regular melt crystallization, the metastable tetragonal form II with 11/3 helix is preferentially formed because of the significant kinetic advantage. However, the most thermodynamically stable modification is the trigonal phase with 3/1 helix [17,18,19,20,21,22,23,24,25]. This means that the initial tetragonal form II crystallites spontaneously transform into the more stable trigonal phase, referred to as form I. After the occurrence of such a solid II–I phase transition, the melting temperature rises and some mechanical properties such as the modulus also improve significantly [26,27,28,29,30,31], but the sample volume decreases [32,33]. Although PB-1 may also crystallize into orthorhombic form III with 4/1 helix directly from a dilute solution [34], the phase transition from form II to form I is a crucial issue in the processing and use of polybutene-1 materials. The practical kinetics of the phase transition is slow, such that the production cost increases. Therefore, control of the phase transition is crucial for the development of polybutene-1 materials.

It has been found that the phase transition can be regulated by the incorporation of certain comonomers. For example, Zheng et al. synthesized a series of butene-1/4-methyl-1-pentene (4M1P) random copolymers. When the 4M1P incorporation exceeds 3.4 mol%, 4M1P branches are high enough to completely impede the II–I phase transition, even when the aging time is as long as four months [35]. The phase transition can also be accelerated by physical and chemical approaches, such as high pressure [36], pressurized CO_2_ [37,38,39], external or thermal stress [40,41,42,43], and even co-units [29,31,44,45,46]. Qiao et al. found that the PB-1 samples were isothermally crystallized into metastable form II crystalline modification followed by annealing at a lower temperature and subsequently at a higher temperature to promote the polymorphic transition from form II to form I. They found that annealing at low temperatures benefits nucleation due to the internal stress induced by the unbalanced shrinkage of amorphous and crystalline phases with different thermal expansion coefficients [47]. An et al. synthesized polybutene-1 ionomers and pointed out that ions tend to aggregate to form clusters as additional intercrystalline links to enhance II–I phase transition [48]. In addition to ionic interaction, polar groups have been reported to be usable for the regulation of crystallization behaviors. Gupta et al. recently found that the introduction of hydroxyl groups into polypropylene and polyethylene could increase the nucleation density [7]. Thus, it is conjectured that polar hydroxyl groups could provide a potential approach to tuning phase transition. However, to the best of our knowledge, there has been no study of functionalized polybutene-1 with hydroxyl groups yet, so the correlation between II–I phase transition with the hydroxyl groups remains unknown.

In this work, butene-1 copolymers with polar hydroxyl groups were successfully prepared and the influences of hydroxyl groups on crystallization and phase transition were explored with differential scanning calorimetry. It is interesting to find that the presence of hydroxyl groups, especially at a high incorporation of 1.31 mol%, significantly accelerated the phase transition kinetics. For nucleation in the phase transition, the optimal temperature was ‒10 °C, independent from the incorporation. Moreover, the accelerating effect of hydroxyl groups on the kinetics of II–I phase transition increased with the decreasing crystallization temperature of form II crystallites.

## 2. Experimental Section

### 2.1. Materials

**Scheme 1:**
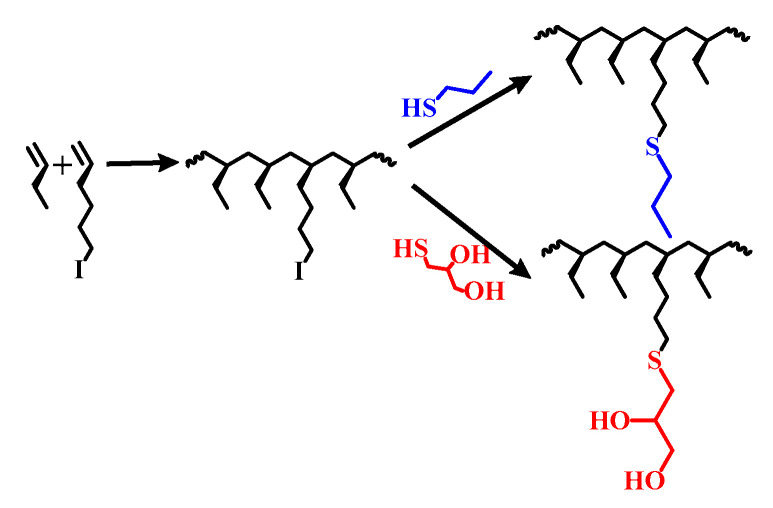
The synthetic routes of the butene-1/6-iodo-1-hexene copolymers and functional polybutene-1.

The polymers studied in this work were functionalized polybutene-1 with and without hydroxyl groups. All synthetic experiments involving air- and/or moisture-sensitive compounds were carried out in a MBraun glovebox, filled with nitrogen and using the standard Schlenk technique. The diemethyl (pyridylamido) hafnium pre-catalyst and the 6-iodo-1-hexene comonomers were synthesized according to [49,50]. The toluene used was purified by an Etelux solvent treatment system. Butene-1 gas and ultrahigh-purity nitrogen were obtained from Tianjin Liufang Industrial Gas Distribution Co., Ltd. (Tianjin, China). Triisobutylaluminum (Al(*^i^*Bu)_3_, 1.1 mol/L in toluene), the tritytetrakis (pentauorophenyl) borate ([Ph_3_C] [B(C_6_F_5_)_4_]) cocatalyst, trimethylamine, 1-propanethiol, and monothioglycerol were purchased from J&K China Chemical, Ltd. (Beijing, China) and used without further purification. The synthetic routes of the butene-1/6-iodo-1-hexene copolymers and functional polybutene-1 are shown in Scheme 1.

### 2.2. Copolymerization of Butene-1 with 6-Iodo-1-Hexene

The copolymers were synthesized following the method described in [48]. The obtained copolymers needed to be redissolved in toluene at 100 °C and precipitated in ethanol dropwise to wash off the excess comonomers and the residual catalyst decomposition products. Finally, the butene-1/6-iodo-1-hexene copolymers were dried in a vacuum oven at 40 °C for 24 h. The incorporation of comonomers can be measured by ^1^H NMR (Figure S1a).

### 2.3. Synthesis of Functionalized Polybutene-1

The obtained copolymers were dissolved in 30 mL of decalin at 90 °C. After cooling to room temperature, *N*,*N*-dimethylformamide, thioglycerol, and trimethylamine were added to the reaction system. The mixture was reacted at 70 °C for 50 h. The obtained butene-1 copolymers with hydroxyl groups were precipitated out by pouring the reaction mixture into a large amount of ethanol. The polymers were washed repeatedly with acetone. After drying under a vacuum at 40 °C for 24 h, the functionalized polybutene-1 with hydroxyl groups was obtained [51]. In addition, thioglycerol was substituted by 1-propyl mercaptan in the same way to prepare the reference polymer with a similar branch length but no hydroxyl group (Scheme 1). ^1^H NMR results show that the reaction was complete (Figure S1b,c). The obtained polymers with and without hydroxyl groups were referred to as PBHY-2 and PBHY-0, respectively, for which the number in the sample code corresponds to the number of hydroxyl groups in each branch (see Table 1).

### 2.4. Methods

The nuclear magnetic resonance (^1^H NMR) characterizations were performed with a Bruker 400 MHz spectrometer (Karlsruhe, Germany). All samples were measured at 120 °C with C_2_D_2_Cl_4_ as a solvent. The molecular weights and molecular weight distributions of the copolymers were measured at 150 °C in 1,2,4-C_6_Cl_3_H_3_ versus polystyrene standards using an Agilent PL-GPC 220 high-temperature gel permeation chromatography, as summarized in Table 1.

The differential scanning calorimetry (DSC) experiments were carried out with Discovery DSC25 (TA instrument, DE, USA) under a nitrogen atmosphere. The sample was first annealed at 180 °C for 10 min to erase the thermal history. Then, both the dynamic cooling crystallization and the isothermal crystallization were studied. The dynamic cooling experiments were carried out at a rate of 10 °C/min. Considering the various crystallization abilities of the copolymers, broad crystallization temperature ranges of 80‒95 °C and 65‒77 °C were chosen for 0.38 and 1.31 mol%, respectively. In the isothermal experiments, a much higher cooling rate of 50 °C/min was employed to prevent crystallization before reaching the desired temperature.

**Scheme 2:**
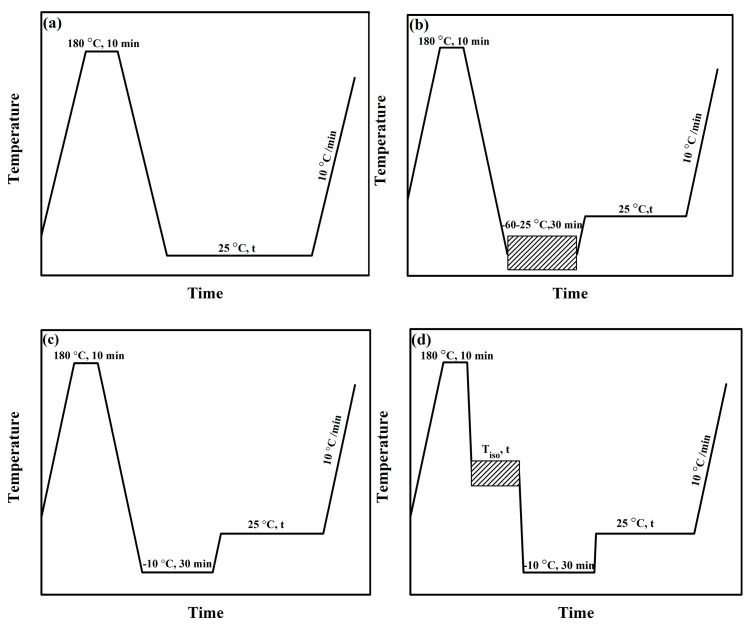
Schematic illustrations of the thermal protocols applied in this work to study (**a**) phase transition kinetics at room temperature, (**b**) dependence of phase transition on nucleation temperature, (**c**) phase transition kinetics with the optimized nucleation step, (**d**) dependence of phase transition on the form II crystallization temperature.

Moreover, DSC was employed to apply different thermal protocols to prepare the crystallites for subsequent phase transition. Scheme 2a shows the formation of form II with the DSC dynamic cooling protocol and the transformation from form II to form I at room temperature (25 °C). Scheme 2b shows that the formation of form II is the same as in Scheme 2a, followed by annealing at a low temperature (*T*_low_) between ‒60 °C and 25 °C for 30 min and sequentially at 25 °C for 50 or 120 h (for 0.38 and 1.31 mol%, respectively). With respect to Scheme 2b, Scheme 2c shows that annealing at low temperature is fixed at ‒10 °C for 30 min and that at high temperature is fixed at 25 °C for different durations. Scheme 2d shows that form II was obtained by isothermal crystallization, which then was annealed using the protocol in Scheme 2c. The last step of all the thermal protocols was heating from 25 °C to 180 °C at 10 °C/min to assess the contents of transformed form I.

The polarized optical microscopy experiments were performed with a Nikon ECLIPSE LV100N POL microscope (Tokyo, Japan) equipped with an external hot stage. After cleaning the thermal histories at 180 °C, the polymers were cooled to the desired isothermal temperatures to monitor spherulite growth: 95 °C and 77 °C for the incorporations of 0.38 and 1.31 mol%, respectively.

The wide-angle X-ray diffraction (WAXD) method was employed to verify that the crystal modification after cooling at 10 °C/min to 25 °C was form II. The WAXD measurements were carried with the Bruker D8 Discovery X-ray setup (Karlsruhe, Germany), where the wavelength of X-ray used was 1.54 Å. The sample-to-detector distance was 56 mm. The measurement time of each frame was 1 min.

## 3. Results and Discussion

### 3.1. Influence of the Hydroxyl Groups on Crystallization

**Figure 1:**
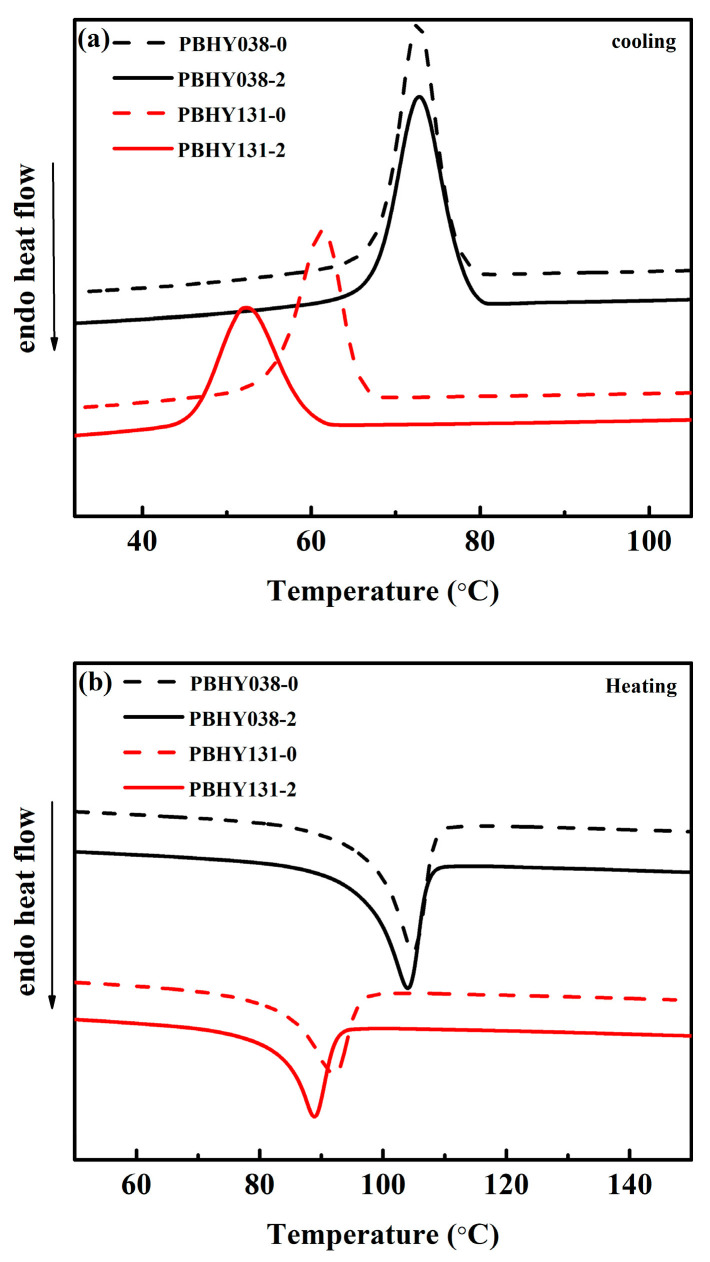
Differential scanning calorimetry (DSC) (**a**) cooling and (**b**) heating curves of the butene-1 copolymers with and without hydroxyl groups.

Figure 1 shows the DSC cooling and heating curves of functional polybutene-1 with hydroxyl groups for two distinct incorporations of 0.38 and 1.31 mol%. It can be seen that, when the incorporation is only 0.38 mol%, the cooling crystallization temperatures are very close between the copolymers with and without hydroxyl groups. As the incorporation increased to 1.31 mol%, the cooling crystallization temperature of PBHY131-0 with more branches decreased with respect to the aforementioned copolymer PBHY038-0. Interestingly, the crystallization of corresponding copolymer PBHY131-2 with hydroxyl groups further decreased to 52.3 °C versus 61.3 °C in PBHY131-0 for the dynamic cooling process at 10 °C/min. Moreover, although the crystallization kinetics may vary due to the incorporation of branches and hydroxyl groups, the crystallites generated were always tetragonal form II, which is the kinetically favored phase of polybutene-1 (WAXD results given in Figure S2).

**Figure 2:**
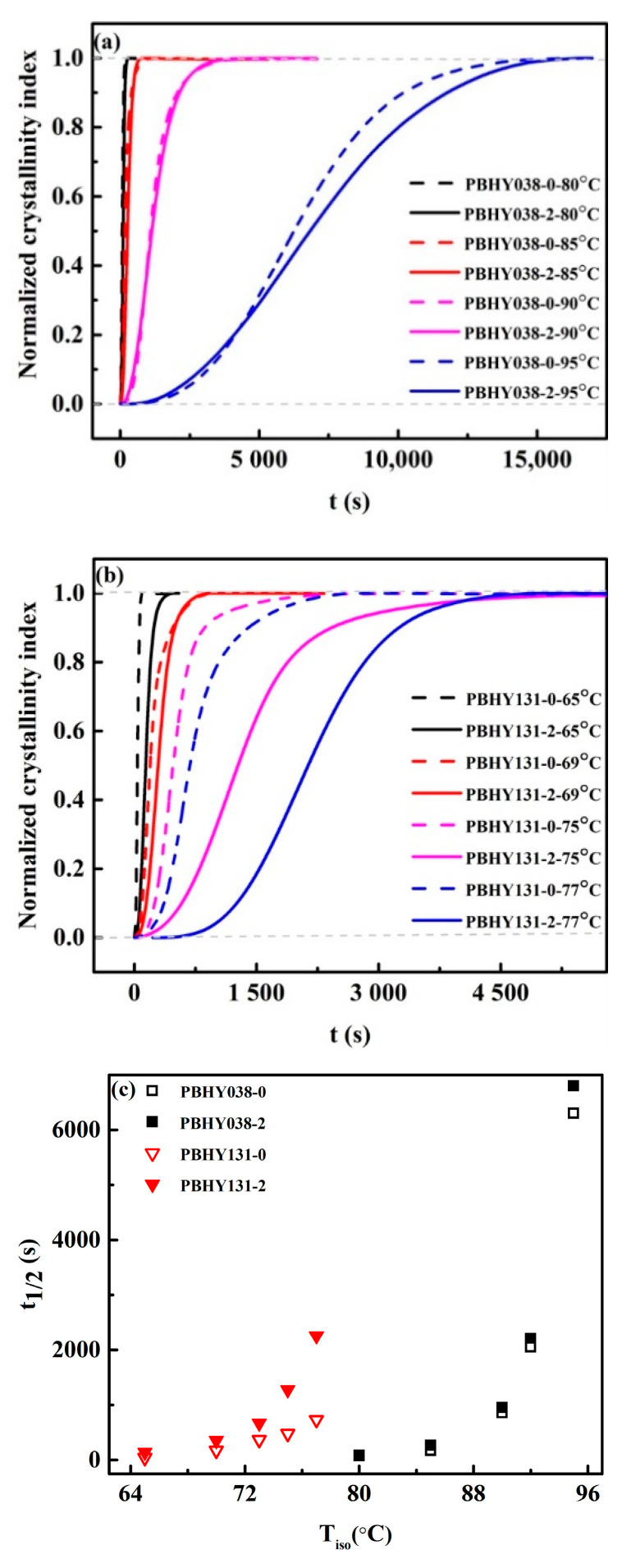
The DSC isothermal curves of copolymers with incorporations of (**a**) 0.38 and (**b**) 1.31 mol% at different temperatures. (**c**) The determined half-crystallization time (*t*_1/2_) as a function of isothermal temperatures.

**Figure 3:**
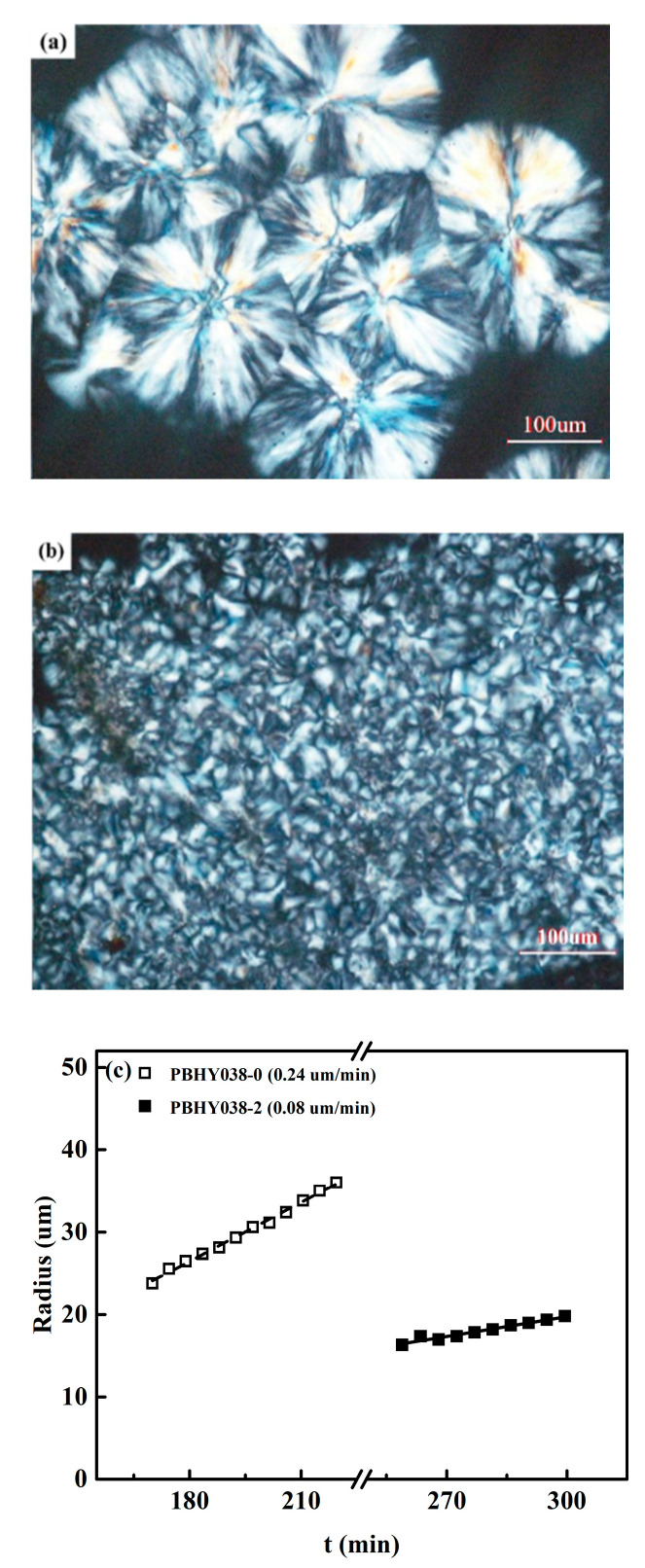
Representative POM images of (**a**) PBHY038-0 without hydroxyl groups and (**b**) PBHY038-2 with hydroxyl groups at a crystallization temperature of 95 °C. (**c**) The change in spherulite radius measured with POM during isothermal crystallization at 95 °C and the determined growth rates.

In addition to the dynamic cooling experiments, the isothermal crystallization behaviors were explored at higher temperatures, which were chosen within 80‒95 °C and 65‒77 °C for incorporations of 0.38 and 1.31 mol%, respectively. In Figure 2a,b, the solid and dashed lines present the crystallization of copolymers with and without hydroxyl groups, respectively. It was observed that, for the low incorporation at 0.38 mol%, the presence of hydroxyl groups seemed not to obviously influence the crystallization kinetics at the relatively low temperature of 80 °C, consistent with the comparable cooling crystallization that occurs within approximately 60–80 °C. However, slowed crystallization kinetics by hydroxyl groups is observed with an elevation in isothermal temperature and an increase in incorporation. Figure 2c displays the quantified half-crystallization time (*t*_1/2_) as a function of isothermal temperature. For example, the presence of hydroxyl groups at the incorporation of 1.31 mol% can increase *t*_1/2_ from 730 to 2250 s for the isothermal crystallization at 77 °C. It is known that by both the nuclei density and the linear growth rate contributed to the crystallization kinetics [52,53,54,55]. In order to gain insight into the variation in crystallization kinetics with the hydroxyl groups, the crystallization process was monitored by polarized optical microscopy. From Figure 3a,b, it can be seen that the nuclei density of PBHY038-2 was significantly higher than that of PBHY038-0, which was consistent with the results observed for 1.31 mol% (Figure S3). However, the linear growth rates that were determined from the corresponding POM images decreased from 0.24 μm/min in PBHY038-0 to 0.08 μm/min in PBHY038-2. It was indicated that, for 0.38 mol% incorporation, the distinct crystallization kinetics of PBHY038-2 with respect to PBHY038-0 is because of the increase in nuclei density and the decrease in linear growth rate. In this case, it could be inferred that the much slower crystallization kinetics of PBHY131-2 is probably due to the further increase in nuclei density not compensating for the decrease in linear growth rate.

### 3.2. Influence of the Hydroxyl Groups in Phase Transition

**Figure 4:**
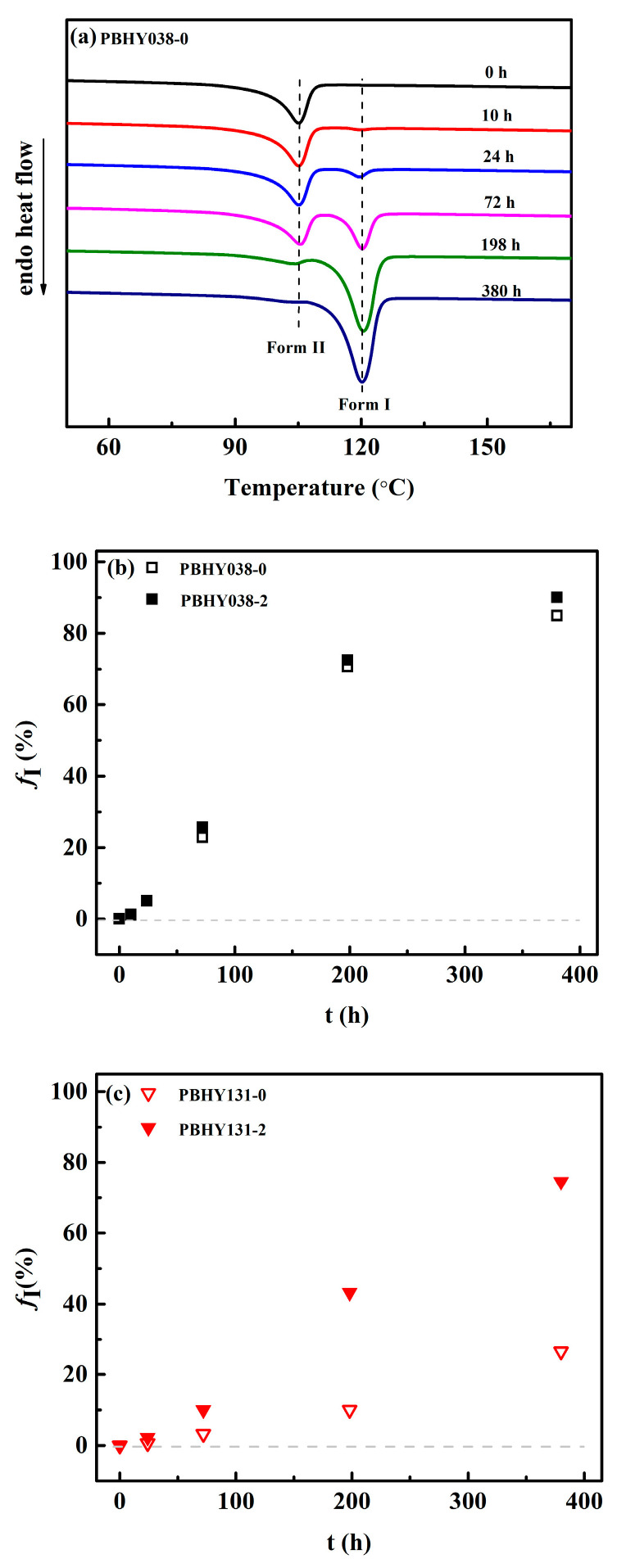
(**a**) DSC melting curves of PBHY038-0 and the quantified fraction of transformed form I after aging at 25 °C for different durations in (**b**) PBHY038 and (**c**) PBHY131. The thermal protocol was illustrated by Scheme 2a.

Although the crystallization temperatures were dependent on the incorporation and type of functionalized groups, the resulting crystallites were all of thermally metastable tetragonal form II (Figure S2), which tends to spontaneously transform into the more stable trigonal form I. To study the influence of hydroxyl groups on the phase transition from tetragonal form II to trigonal form I, the generated form II crystallites were aged at 25 °C for various durations and then heated to determine the fraction of transformed form I in the total crystallites, since form I has a higher melting temperature than form II. Figure 4a first shows the DSC heating curves of polymer PBHY038-0 after annealing at 25 °C for different times. It was seen that, without annealing, only a single endothermic peak of form II was observed in the heating curve. However, after annealing for 10 h, one additional distinct endothermic peak appears, which corresponds to melting of the transformed form I [48,56]. As the annealing period was further extended, the endothermic peak of the transformed form I increased whereas that of the residual form II decreased correspondingly. The fraction of transformed form I can be quantified from the DSC results with the following equation: $f_{I}=\frac{A_{I}/\Delta H_{I}^{0}}{A_{I}/\Delta H_{I}^{0}+A_{\mathrm{II}}/\Delta H_{\mathrm{II}}^{0}}$, where $A_{I}$ and $A_{\mathrm{II}}$ represent areas of endothermic peaks during melting of form I and form II, respectively, and $\Delta H_{I}^{0}$ and $\Delta H_{\mathrm{II}}^{0}$ are the crystal melting enthalpies of pure forms I and II, 141 and 62 J/g, respectively [28]. Figure 4b,c compare the evolution of fractions of transformed form I with and without hydroxyl groups for two distinct incorporations of 0.38 and 1.31 mol%, respectively. When the incorporation is low, only 0.38 mol%, the kinetics of the phase transition with hydroxyl groups are comparable with those without hydroxyl groups (Figure 4b). As the incorporation increased to 1.31 mol%, the II–I phase transition with hydroxyl groups is higher than that without hydroxyl groups and the difference increases with the phase transition. It was indicated that the presence of hydroxyl groups could accelerate phase transition for the high incorporation of 1.31 mol%.

### 3.3. The Relationship between Phase Transition and Transition Temperatures

**Figure 5:**
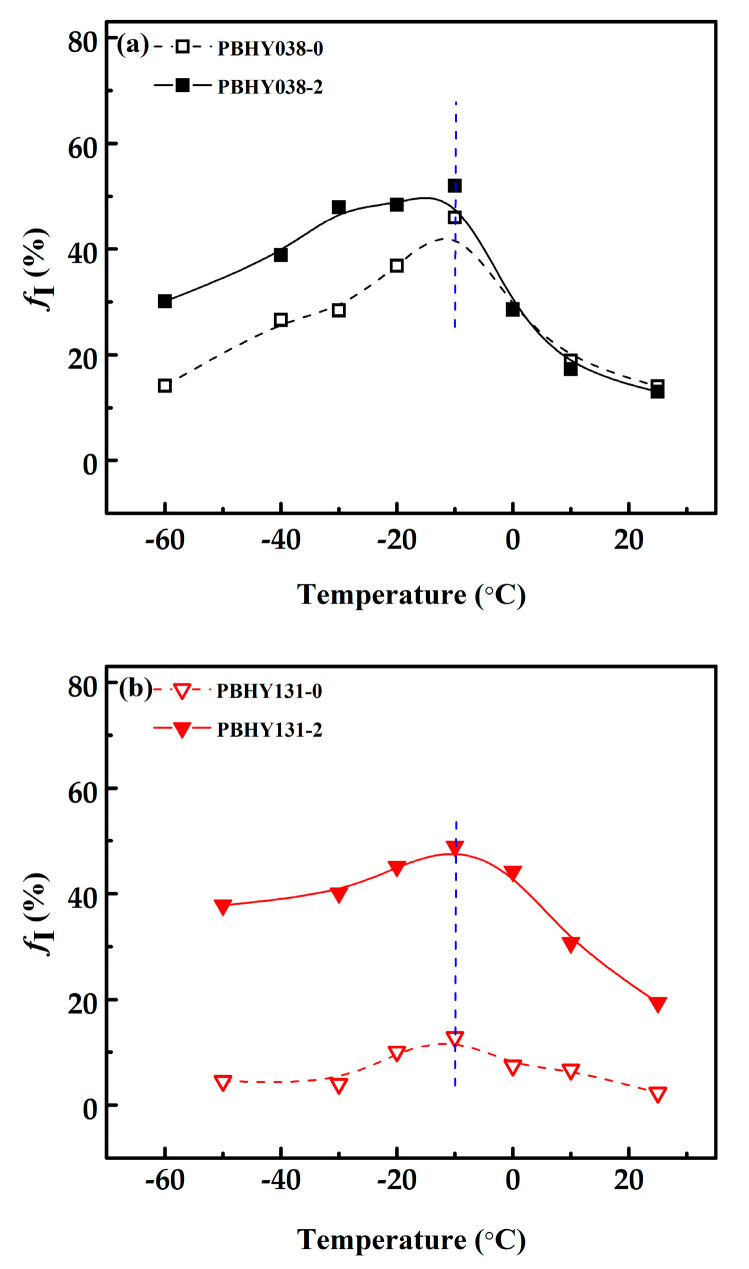
Fraction of transformed form I as a function of annealing temperature in (**a**) PBHY038 and (**b**) PBHY131. The thermal protocol was illustrated by Scheme 2b.

It has been recognized that solid II–I phase transition is implemented as a two-step process, triggered by nucleation and subsequently implemented by the growth of form I within the initial lamellae [57,58]. Moreover, it has been reported that the kinetics of II–I phase transition is determined by the nucleation step [56,59,60,61]. In this case, the kinetics of the phase transition is strongly dependent on the nucleation temperature. The lowered temperature could increase the temperature difference between crystallization and nucleation, which increases the internal thermal stress originating from the various thermal expansion coefficients between the crystal and amorphous regions, and might also decrease the critical nuclei size for the newly appeared phase. To examine the relationship between phase transition kinetics and the transition temperature, the form II crystallites were cooled to various temperatures for a fixed duration of 30 min to allow for nucleation and were subsequently aged at 25 °C for the transition. Due to the different kinetics, the transition periods were chosen as 50 and 120 h for 0.38 and 1.31 mol%, respectively. From Figure 5, it can be clearly seen that, irrespective of the presence of hydroxyl groups, the maximum peak of transformed form I is observed at ‒10 °C, indicating an identical optimal temperature for nucleation. In addition, this optimal temperature remains unchanged when increasing the incorporation from 0.38 to 1.31 mol%, which is the same as for the nucleation temperature in polybutene-1 homopolymer [56].

**Figure 6:**
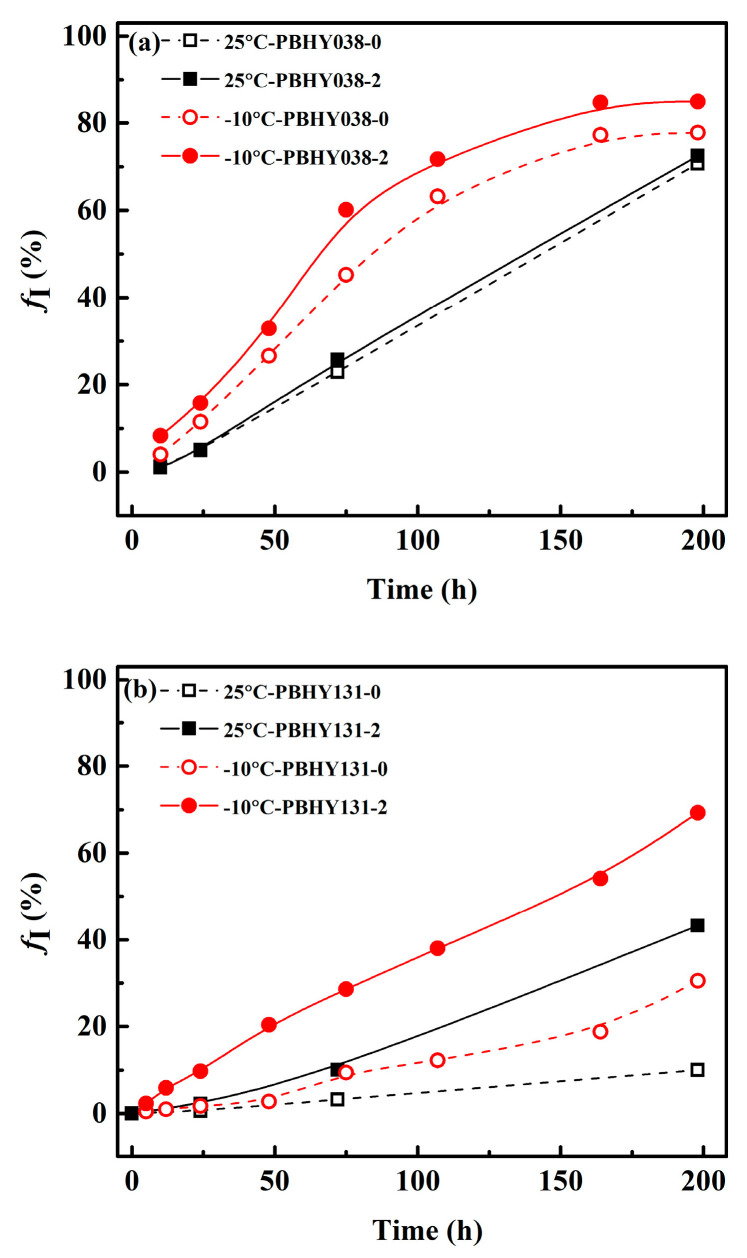
The fractions of the form I transformed at room temperature after initial nucleation at −10 °C for 30 min in (**a**) PBHY038 and (**b**) PBHY131. The thermal protocol was illustrated by Scheme 2c.

With the aforementioned optimal nucleation temperature of ‒10 °C, the solid II–I phase transition was monitored and the evolution of the transformed form I was as shown in Figure 6. We notice that, compared to the phase transition behavior at 25 °C, the annealing at ‒10 °C for 30 min in advance can greatly accelerate the phase transformation. Moreover, the accelerating influence of hydroxyl groups in II–I phase transition was also confirmed for the relatively low incorporation of 0.38 mol%.

### 3.4. The Relationship between Crystallization Temperature and Phase Transition

**Figure 7:**
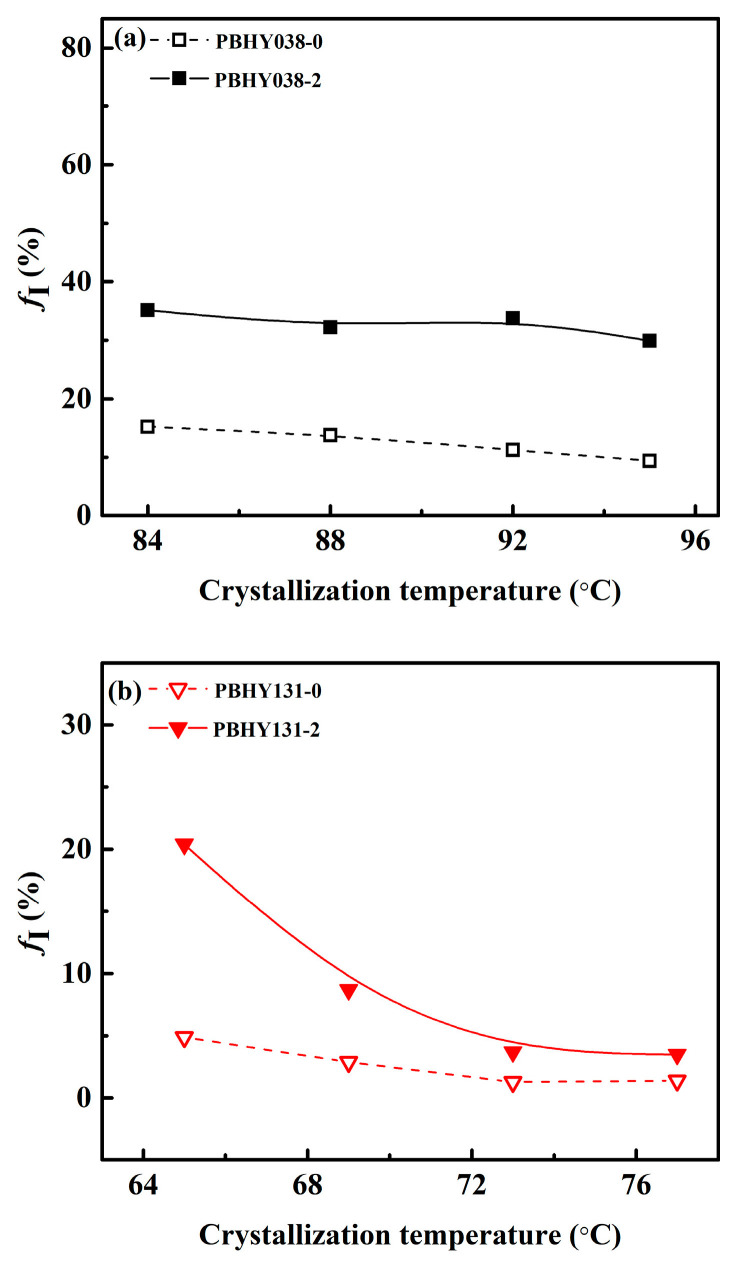
The fractions of transformed form I in copolymers of (**a**) PBHY038 and (**b**) PBHY131 prepared at different isothermal crystallization temperatures. The thermal protocol was illustrated by Scheme 2d.

The above results focus on the influence of transition conditions on the transition kinetics. It should be noticed that the structural characters of the initial form II that are transformed are strongly dependent on the crystallization process from amorphous melting to the ultimate solid. It was recently reported that the temperature used to prepare the initial crystallites not only determines the transition conditions to influence the internal stress but may also vary the number of intercrystalline links, which plays an important role in the stress transport between lamellae [47]. Note that the initial form II crystallites studied in the above sections were all prepared by a cooling process, where there is a difference in the crystallization temperature between PBHY131-0 and PBHY131-1 (Figure 1). To know the dependence of II–I transition on the crystallization temperature, the form II crystallites to be transformed were prepared at various temperatures. Figure 7 shows the fractions of transformed form I as a function of isothermal temperature of form II preparation. It was observed that the fraction of transformed form I increases slightly with decreasing temperature for a low incorporation of 0.38 mol% (PBHY038-0 and PBHY038-2, shown in Figure 7a) and a high concentration of 1.31 mol% without a hydroxyl group (PBHY131-0, shown in Figure 7b). However, PBHY131-2 with substantial hydroxyl groups shows increased transformed form I when lowering the form II preparation temperature. This means that the influence of hydroxyl groups on II–I transition kinetics becomes weaker at elevated temperatures. The increase in transition kinetics with lowering crystallization temperature was also observed in polybutene-1 ionomers with the physical ion aggregates [48]. It was indicated that the hydroxyl groups may form some hydrogen-bonded nanoclusters, similar to ion aggregates, which act as additional physical crosslinks to enhance internal stress to the lamellae for form I nucleation within form II. In summary, in the presence of polar hydroxyl groups, the solid II–I phase transition could be more efficiently accelerated by lowering the crystallization temperature of preparing form II.

## 4. Conclusions

In this work, functionalized polybutene-1 copolymers with hydroxyl groups at distinct incorporations of 0.38 and 1.31 mol% were synthesized. The crystallization kinetics of tetragonal form II and the subsequent phase transition into trigonal form I were studied with differential scanning calorimetries. The influence of the hydroxyl groups on crystallization and phase transition is dependent on the incorporation. For a low incorporation of only 0.38 mol%, the introduced hydroxyl groups do not significantly affect the kinetics of the dynamic cooling crystallization or the isothermal crystallization at temperatures below 85 °C, which are associated with increased nucleation density but a decreased growth rate. Differently, when the incorporation was increased to 1.31 mol%, the presence of hydroxyl groups significantly decreased the crystallization temperature.

More importantly, the solid phase transition from form II to I could be accelerated by the presence of hydroxyl groups. The results show that the influence of hydroxyl groups on II–I phase transition is increased when increasing the incorporation from 0.38 to 1.31 mol%. In addition, the phase transition exhibits a nonmonotonic dependence on temperature and the optimal nucleation temperature to obtain a maximum transition degree at ‒10 °C, independent from the content of hydroxyl groups. Furthermore, the accelerating influence of hydroxyl groups on phase transition further increases when lowering the crystallization temperature of preparing form II.

## Figures and Tables

**Table 1:** Molecular characteristics of the butene-1 copolymers.

Sample Code	Incorp. (mol%)	Number of Hydroxyl Groups in Each Branch	*M*_w_ (10^5^ Da)	*M*_w_/*M*_n_
PBHY038-0	0.38	0	6.9	2.02
PBHY038-2	0.38	2	6.9	2.02
PBHY131-0	1.31	0	5.2	2.23
PBHY131-2	1.31	2	5.2	2.23

## Supplementary Materials

The following are available online at https://www.mdpi.com/article/10.3390/polym13081315/s1, Figure S1: NMR results; Figure S2: WAXD results; Figure S3: POM results.
