# Supplementary material for: The II–I Phase Transition Behavior of Butene-1 Copolymers with Hydroxyl Groups
Source: Polymers (Basel). 2021 Apr 16;13(8):1315. doi: 10.3390/polym13081315 (PMC8074023; doi:10.3390/polym13081315)
Supplement: Supplementary file 1 [file polymers-13-01315-s001.zip › polymers-1172774-supplementary.pdf]

polymers

MDPI

Supporting Information

The II–I Phase Transition Behavior of Butene-1 Copolymers with Hydroxyl Groups

Yuanyuan Li [1], Tao Li [1], Wei Li [1], Yahui Lou [1], Liyuan Liu [2,*] and Zhe Ma [1,*]

[1] Tianjin Key Laboratory of Composite and Functional Materials and School of Materials Science and Engineering, Tianjin University, Tianjin 300072, China; yyli@tju.edu.cn (Y.L.); litao0416@tju.edu.cn (T.L.); weili_wq@tju.edu.cn (W.L.); yhlou@tju.edu.cn (Y.L.)

[2] Center for Terahertz Waves and College of Precision Instrument and Optoelectronics Engineering, Tianjin University, Tianjin 300072, China

* Correspondence: lyliuma@tju.edu.cn (L.L.); zhe.ma@tju.edu.cn (Z.M.)

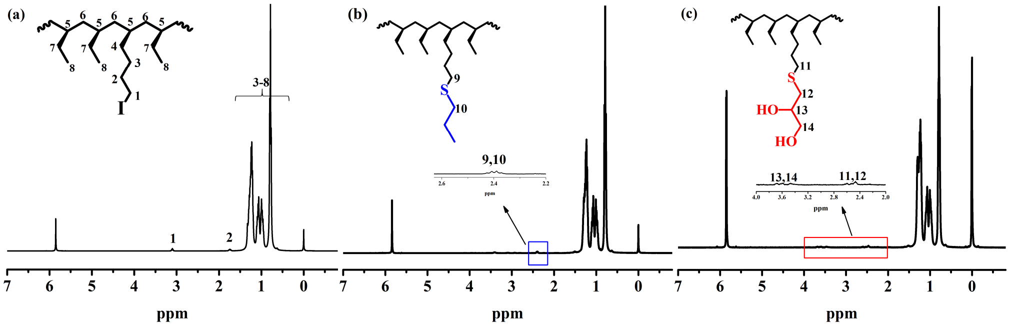

Figure S1. ^{1}H-NMR spectra of (**a**) butene-1/6-iodo-1-hexene copolymer, the functionalized copolymer (**b**) without hydroxyl groups and (**c**) with hydroxyl groups.

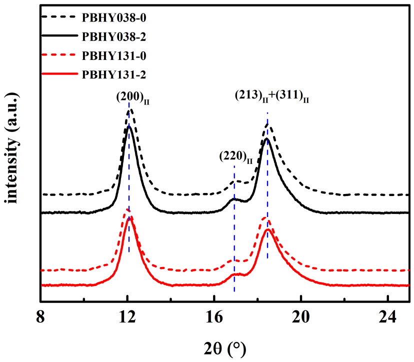

Figure S2. The wide angle X-ray diffraction (WAXD) results of the functionalized butene-1 copolymers after the dynamic cooling experiments.

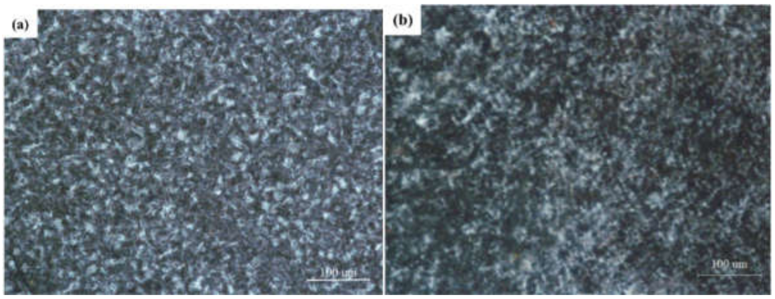

Figure S3. The polarized optical micrographs of butene-1 copolymer (**a**) PBHY131-0 without hydroxyl groups and (**b**) PBHY131-2 with hydroxyl groups after the isothermal crystallization at 77 °C.
